# Ovariotomy in Battey’s Infirmary

**Published:** 1885-09

**Authors:** 


### Editorial.

### OVARIOTOMY IN BATTEY'S INFIRMARY.

We are satisfied that a large number of our readers have no idea of the magnitude of this special work done by Dr. Robert Battey, of Rome, and a detailed description of it will, we are sure, be of interest to them.

While in Rome some weeks since, we visited this infirmary, which is located on an elevation on South street, overlooking the Etowah river. It consists of seven neat cottages of five rooms each, with beautiful grounds surrounding them. The place presents quite an attractive appearance. The furniture is plain, but neat. The bedsteads are brass with woven wire mattresses.

Since January 1st of this year, Dr. Battey has averaged one ovariotomy each week. These tumors, after being removed, are preserved in arsenic, stuffed and carefully put away. In the collection we noticed twenty of very large size, the largest one weighing, when removed, fifty-six pounds.

The success that has followed the removal of these large tumors has been something wonderful—not one of the cases having died. The Doctor attributes this success, in a great measure, to the antisceptic precautions that he observes with every case.

The operation we witnessed was in a patient 32 years old. She had been remarkably stout and healthy up to September, 1884, when she noticed an enlargement of the abdomen. This continued to enlarge rapidly, and very soon she was treated for

"dropsy," with drastic purgatives until her general health began to fail, without any diminution of the enlargement. Since December 1st she has not menstruated. She was admitted to the infirmary four days prior to the operation, and the only preparatory treatment given her was a compound cathartic pill, given two days previous to the operation and an enema of warm water given on the morning of the operation. She was not allowed any food the night previous to the operation or the morning of it. It will be seen that at the time of the operation the stomach was empty.

The patient was placed on a short table with her feet resting in a chair, and was etherized with Squibb's ether by Dr. George R. West, one of the assistants.

The strictest antisceptic precautions were observed throughout. From the time the patient was etherized and the abdomen exposed, until the operation was completed, a spray of carbolic acid 1 to 40 was kept constantly playing on her from a large steam atomizer.

The incision was made in the median line with an ordinary scalpel, down to the sheath of the rectus muscle, when the remainder of the tissues down to the peritoneum were divided on a grooved director with scissors. When the peritoneum was reached, all cutting was suspended until the bleeding points had been secured by forceps, after which the peritoneal sac was opened and a trocar thrust into the tumor, when four and a half gallons of fluid almost as black as tar escaped. There were numerous adhesions to the abdominal walls which were broken up with the hand, after which the pedicle was ligated with a stout silk ligature and the tumor removed. It weighed 47 pounds. On account of the adhesions there was considerable oozing of blood into the peritoneal cavity. This was arrested by packing the cavity with sponges, after which the cavity was thoroughly washed out with warm water and the wound closed with silk sutures. No drainage tube was used. The dressing consisted of a small piece of old linen spead with carbolic cerate placed over the wound, with a compress of uncarded cotton. Over this was placed an abdominal bandage of flannel, which completed the dressing. The entire operation, from the beginning until the patient was removed from the table, consumed one hour and five minutes.

The after-treatment, as we are informed by Dr. Battey, consists chiefly in doing nothing. He says that the only thing required is an occasional anodyne enema, and a great many do not require that. Dr. Battey is ably assisted in this work by his son, Dr. Henry H. Battey, Dr. George R. West, Mrs. Battey and his two students, Mr Harry Huzza, of this city, and Mr. Glover. Mrs. Battey has entire charge of the nursing and general management of the patients after the operations, and, in our opinion, it is to her good nursing and kind and gentle treatment, more than to the strict antiseptic precautions, that the great success of the operations is due.

